# Tracking the Small with the Smallest – Using Nanotechnology in Tracking Zooplankton

**DOI:** 10.1371/journal.pone.0013516

**Published:** 2010-10-29

**Authors:** Mercy Lard, Johan Bäckman, Maria Yakovleva, Bengt Danielsson, Lars-Anders Hansson

**Affiliations:** 1 Institute of Biology, Limnology, Lund University, Lund, Sweden; 2 Department of Infectious Diseases and Medical Microbiology, Lund University, Lund, Sweden; 3 Department of Pure and Applied Biochemistry, Lund University, Lund, Sweden; Monash University, Australia

## Abstract

A major problem when studying behavior and migration of small organisms is that many of the questions addressed for larger animals are not possible to formulate due to constraints on tracking smaller animals. In aquatic ecosystems, this problem is particularly problematic for zoo- and phytoplankton, since tracking devices are too heavy to allow the organism to act naturally. However, recent advances in nanotechnology have made it possible to track individual animals and thereby to focus on important and urgent questions which previously have not been possible to address. Here we report on a novel approach to track movement and migratory behavior of millimeter sized aquatic animals, particularly *Daphnia magna*, using the commercially available nanometer sized fluorescent probes known as quantum dots. Experimental trials with and without quantum dots showed that they did not affect behavior, reproduction or mortality of the tested animals. Compared to previously used methods to label small animals, the nano-labeling method presented here offers considerable improvements including: 24 h fluorescence, studies in both light and darkness, much improved optical properties, potential to study large volumes and even track animals in semi-natural conditions. Hence, the suggested method, developed in close cooperation between biologists, chemists and physicists, offers new opportunities to routinely study zooplankton responses to light, food and predation, opening up advancements within research areas such as diel vertical/horizontal migration, partial migration and other differences in intra- and interspecific movements and migration.

## Introduction

Movements and migration of larger animals, like birds and fish, can relatively easily be tracked by devices such as satellite transponders and PIT tagging [1], [2], [3]. However, with respect to small organisms, the tracking methods are far from sufficient and traditional radio-tracking as a method is today not applicable for these organisms. As a consequence, recent advancements in tracking migration of organisms as small as about 10 mm, such as bees and ants, have, despite very advanced techniques, generally lead to rather cruel situations [4]. In some cases the device has even been similar in size to the tracked animal, which rises questions regarding whether or not the recorded behavior and decision making of the tracked animal is merely an artifact, and that the studies may instead produce misleading information rather than improved understanding.

Although the risk that the tracking device may affect the organism can never be excluded, individual tracking has generated important information regarding behavior and causes for movements and migrations in larger animals. Therefore we have here taken advantage of the recent extremely rapid developments within nanoscience including the use of quantum dots typically applied to *in vitro* or *in vivo* biomedical imaging [5], [6], [7], [8], [9], [10], [11]. This development offers opportunities for studies in small-scale biology and ecology, since nanometer sized objects are carried easily even by small (mm-scale) organisms without potential for harm [12], [13]. Moreover, other advantages with nano-sized tracking systems is that they can be used in aquatic ecosystems (i.e. in water) and that the same method can be applied to both consumer, e.g. zooplankton (mm-scale) and their algal, or even bacterial food (µm scale). Although the development of this technique is still in progress, we believe that methods using nanometer tracking devices have the potential to open up new research fields by allowing individual tracking of small organisms. We herein describe a method using nanometer sized fluorescent probes applied to track the zooplankton species *Daphnia magna* and also provide a biocompatibility test quantifying effects of the probes on mortality, behavior, and reproduction.

## Methods

The semiconductor material (CdSe) which comprises the core of the quantum dots used in this experiment has extremely high photostability (i.e. does not bleach easily like other fluorophores), can be excited with a range of different wavelengths, and has a narrow symmetric emission wavelength [11], [14], [15]. This is in part due to an inorganic shell (ZnS) with an outer synthetic polymer coating (amine terminated polyethylene glycol (amino-PEG)), which stabilizes the quantum dot core and makes it water-soluble [16]. Various combinations of these surface coatings bound to the shell can be used to suit a number of applications including forming conjugates with biomolecules [7], [17].

**Figure 1:**
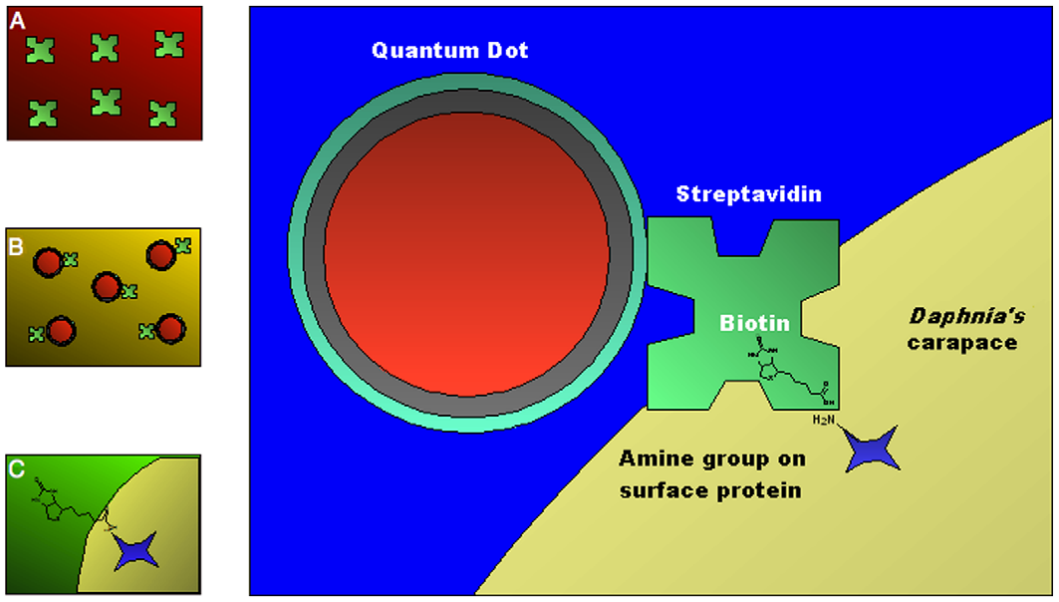
Schematic of labeling process. A) Streptavidin is activated forming a stable ester. B) Streptavidin ester is incubated with quantum dots forming a QD-SA conjugate. C) *Daphnia* are biotinylated via amine containing surface proteins. D) Biotinylated daphnids are placed in water and incubated with the QD-SA conjugate.

In order to properly label the animals with quantum dots (QD) a standard bioconjugation was performed [18], as schematically shown in Fig. 1. High affinity interaction between streptavidin (SA) and biotin (K_a_ approximately 10^−15^ mol/L [19]) was used to form a link between the quantum dots and the *Daphnia*' carapace (Fig. 1). The composition of the shell of *Daphnia* is not precisely known, however, amine containing proteins are likely present on the exoskeleton making it possible to biotinylate them [20]. Streptavidin was attached to quantum dots by standard amine coupling procedure. Briefly, (A) streptavidin from *Streptomyces avidinii* (Sigma Aldrich, Stockholm, Sweden) was dissolved in 10 mM phosphate-buffered-saline (PBS), pH 7.4 to a final concentration of 1 mg/mL. 20 µL of streptavidin was activated by 1∶1 (v/v) mixture of 0.1 M 1-ethyl-3-(3-dimethylaminopropyl)carbodiimide (EDC) (Biacore-GE Healthcare, Piscataway, NJ, USA) and 0.4 M N-hydroxysuccinimide (NHS) (Biacore-GE Healthcare, Piscataway, NJ, USA). The reaction was allowed to proceed for 30 minutes. As a result, a stable ester of streptavidin was formed (Fig. 1A). (B) Activated SA was incubated with quantum dots (1∶2.66 (v/v)) for 30 minutes forming a quantum dot-streptavidin QD-SA conjugate solution (Fig. 1B). For this study two types of QDs were used: 8 µM Qdot® 655 ITK™ amino(PEG) quantum dots and 8 mM Qdot® 585 ITK™ amino(PEG) quantum dots (Invitrogen, Stockholm, Sweden). These quantum dots have emission wavelengths at 655 nm (red) and 585 nm (yellow), respectively. (C) Adult *Daphnia magna* from a culture maintained in the lab since 2008 were incubated with 70 µL of EZ-Link Sulfo-NHS-Biotin (Pierce, Rockford, IL, USA) for 15 min and subsequently washed three times (Fig. 1C). (D) *Daphnia* were then placed into 30 µL of water and incubated with 3 µL of the QD-SA conjugate solution for 15 min, thus forming the non-covalent bond between streptavidin and biotin (Fig. 1D). The animals were washed three times prior to imaging. All handling of *Daphnia* was done with 3 mL disposable pipettes, with end of the tip cut and removed.

**Figure 2:**
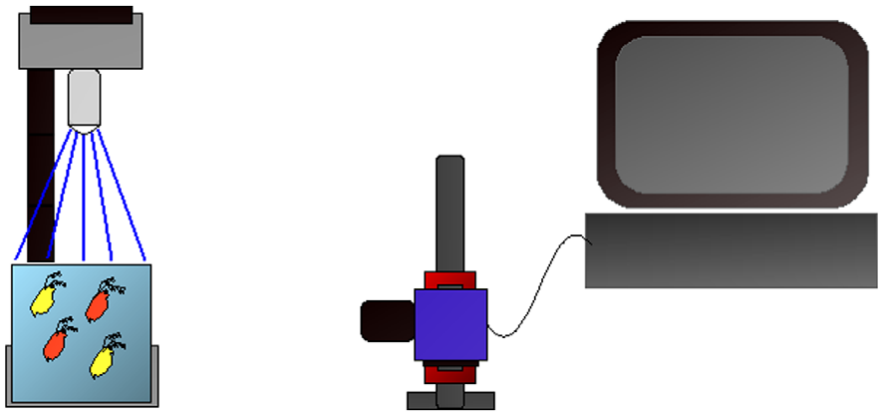
Schematic illustration of the setup used during the experiment. Left: the flexible illumination source is focused on the tank of labeled *Daphnia*. Center: the camera records images of swimming *Daphnia*. Right: the computer is used for processing images and producing videos of tracks.

The quantum dots used in this experiment work like any other fluorescent molecule, in that they must be excited with light of one wavelength in order to emit fluorescence at another longer wavelength. This excitation/emission mechanism is an intrinsic property of semiconductors, and occurs when electrons in the solid material are excited from a lower to a higher energy band (excitation) and then subsequently de-excite (emission) back into a lower energy band giving off photons, which we are able to detect. We selected the particular excitation wavelength based on two restrictions. First, there is a decaying absorption curve for the quantum dots we use, with a peak in the UV region at around 300 nm then dropping to zero near their peak emission wavelengths. Therefore excitation wavelengths are most efficient in the UV-blue region. Another consideration to be made is concerning the photoreceptors present in the *Daphnia*'s compound eye, which are mainly sensitive to four distinct classes of wavelengths including: 348±4 nm, 434±5 nm, 525±4 nm and 608±8 nm, with slight variation between dorsal and ventral ommatidium [21]. Due to this fact we avoided excitation wavelengths within these photoreceptor classes and instead chose a wavelength of 465 nm to effectively excite the quantum dots. Our custom-built illumination source (Fig. 2) was formed from a light emitting diode (LED) (ENFIS, Swansea, UK) with peak emission of 465 nm and full width at half maximum (FWHM) of 25 nm, with measured radiance of 30 W/m^2^ at approximate current setting of 1.5 A. According to the manufacturer's recommendations the LED was secured to an aluminum plate (45×43×3 mm), mounted on an aluminum heat sink (90×75×40 mm) and cooled with a standard computer fan (90×80×20 mm) to avoid overheating. A cylindrical aluminum tube with darkened interior, inner diameter 25 mm and length 40 mm, was secured to the aluminum heat sink and situated over the LED in order to focus the light column. A filter for the LED was used to attenuate unwanted wavelengths coming from the source. We used a Precision Short Pass filter (NT49-818) (Edmund Optics, York, UK) with a rejection wavelength range of 520–610 nm, transmission wavelength range of 250–480 nm, and cut-off wavelength of 500 nm. This filter was placed inside the aluminum tube perpendicular to the light source. A diffuser made of optically opaque and non-fluorescent plastic, 1 mm in thickness, was attached to the end of the tube to produce an equal distribution of light throughout the tank without altering the light spectrum. This entire pairing was then attached, via the aluminum heat sink, to a flexible arm and secured to a stage, allowing easy rotation and adjustment of the light source. A Precision Long Pass filter (NT62-984) (Edmund Optics, York, UK) with rejection range of 200–539 nm, transmission region of 560–1650 nm, and cut-on wavelength at 550 nm was used for the camera lens. The optical density is greater than 4.0 and transmission is greater than 91 percent within the range specified for both filters. Both LED and camera filters have a 25 mm outer diameter. A USB2000 spectrophotometer (Ocean Optics, Ostfildern, Germany) was used to characterize the spectrum after passage through the LED illumination setup and to verify the intended excitation.

A FireWire CCD color camera (DFK21AF04) (The Imaging Source, Bremen, Germany) used for imaging, was mounted perpendicular to the side of the sample chamber under observation. The distance from the edge of the camera lens to the side of the chamber was approximately 150 mm. The CCD camera, with optical resolution of 640×480 pixels per frame, was operated via FireWire connection (PCIe FireWire 1394a) to a desktop PC. A 4 mm lens was used in order to achieve maximum focal depth, approximately 100 mm, inside the test aquarium. The software, Imaging Control 3.0 (The Imaging Source, Bremen, Germany) was used to capture live video files with frame rate set at 7 frames per second. The test aquarium was a glass cube of 100×100×100 mm open at the top. The back and left and right sides of the cube, as well as the edges, were covered in order to eliminate stray light and background fluorescence. The cube was placed on the stage, allowing for maximum illumination of LED into the sample space. Images were taken with the LED source light positioned directly above the cube and perpendicular to the base. The end of the tube with the diffuser attached was approximately 100 mm from the top of the water in the tank (Fig. 2).

**Figure 3:**
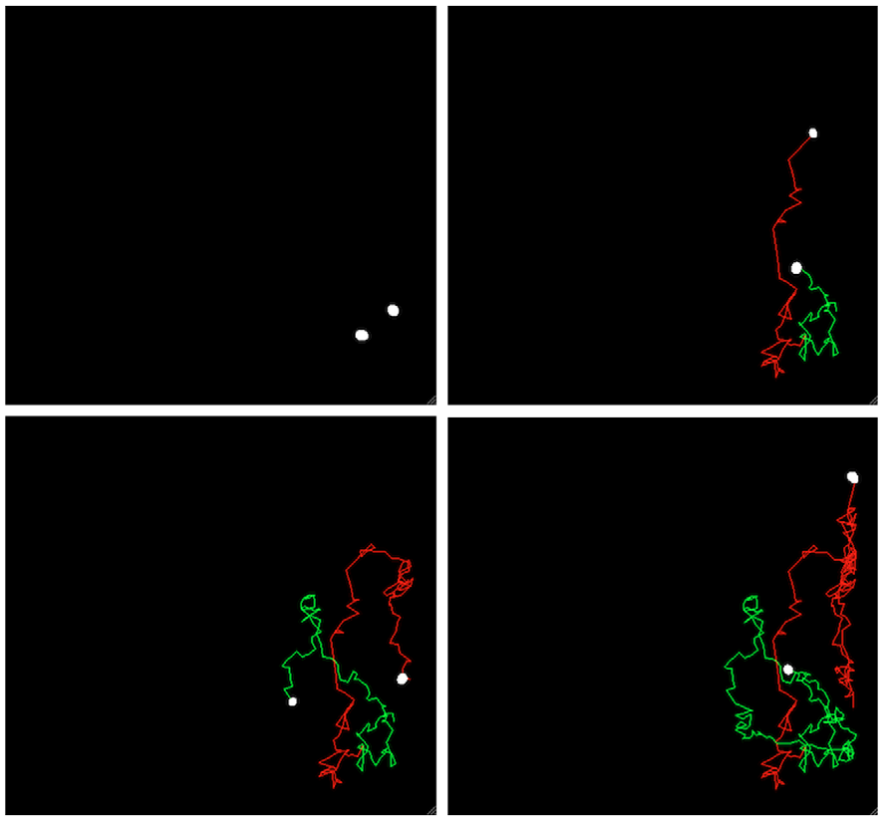
Image sequence of 2-D track of daphnids motion as they swim in the test cube. *Daphnia* are biotinylated and then labeled, each with a separate quantum dot conjugate. Yellow Track: Using Qdot® 585 ITK™ amino(PEG) quantum dots conjugated with streptavidin. Red Track: Using Qdot® 655 ITK™ amino(PEG) quantum dots conjugated with streptavidin.

All recordings were made in complete darkness, with only light from the LED source (465 nm) used to illuminate the test cube. After video capture all files were processed with ImageJ 1.43 image viewing software. A 2-D track was made from the motion of the daphnids in the glass aquarium, using MATLAB version R2009b (Fig. 3). The track was achieved from modifications of a source code previously written for particle tracking. A sequence of frames from the daphnid' position in the tank is shown in Fig. 3, with a time progression from left to right in a period of 37 seconds. The two tracks show one yellow and one red trace each of *Daphnia* swimming in the tank. From these tracks position at different times and at different treatments, as well as distance traveled can be analyzed using MATLAB image analysis.

In order to test the possible toxicity of the chemicals and nanoparticles used on the animals we conducted an assay with treated and non-treated animals. Ten adult *Daphnia* from the same population, each with a clutch present, were isolated in a glass jar with 250 mL from their source water, 250 mL tap water from a copper free source, and 250 mL of *Scenedesmus sp.* green algal culture. Twenty of the offspring hatched within 24 hrs were removed and used for the assay. The neonates were allowed to mature for eight days, prior to treatment, in individual 100 mL glass jars with a mixture of 50 mL source water, 30 mL tap water from a copper free source, and 20 mL of green algae. The non-treated animals were washed and incubated with water in place of chemicals and nanoparticles. First, two *Daphnia* were isolated in one well of a three well glass slide with 70 mL of water for 15 minutes. Then, the animals were washed three times and moved to the adjacent well where they were incubated with 33 mL of water. Finally, the animals were moved to the third well and washed three times with water. The *Daphnia* were then separated back into their individual jars. This was repeated four more times for the control group making a total of 10 individuals. For the test group, identical treatment was used as in the case of filming for biotinylation and incubation with QD-SA conjugates (see *Labeling*). After treatment each of the 10 test animals were placed back into their individual jars. Following this treatment the *Daphnia* were fed three times a week by removing 20 mL of water from their jars and adding in 20 mL of fresh algae. Every 5 days 50 mL of the water from their jars was replaced with copper free tap water. After each hatch the offspring were removed and counted in order to detect any differences in reproductive rates between control and test animals. Counting the offspring was done by carefully removing the adults from test and control group jars with a disposable pipette and then filtering the entire contents of their jars through a 50 mm mesh net individually. The water and algae solution and the adult *Daphnia* were then placed back into their individual jars. The neonates of each animal collected on the net were counted. The reproduction, survival and behavior of adult daphnids were recorded throughout the experiment.

The mean reproductive output, expressed as mean number of offspring per female (n_c_ = n_t_ = 10), was analysed using Students t-test. The same test was used for analysis of survival, expressed as number of days each individual survived.

## Results and Discussion

A major problem when studying small organisms is that many questions, which are easily addressed for larger animals, are not possible to even formulate due to the fact that the tracking devices are too heavy to allow the organism to act naturally. This has indeed hampered research on small organisms, especially in aquatic environments, and has restricted some research areas to resemble black boxes, such as diel vertical/horizontal migration in zooplankton [22], [23] and algae [24], escapes from threats such as ultraviolet (UV) radiation or predation [25], and size-structured dispersal [26]. Hence, one of the major problems for the advancement of research focusing on small organisms is that methodological constraints prevent hypothesis testing. The method we present here allows for tracking individuals and groups with sizes on the millimeter scale. Tracking the labeled organisms using different Quantum dots, or any mixture of different Quantum dot colors (Fig. 3), provides opportunities to individually track a large number of individuals, populations or groups of, for example, differently treated organisms.

Results from our methodological study using bioconjugation of streptavidin with quantum dots and attachment to daphnids via biotin ligands show that daphnids can be tracked in a 2-D plane in dark conditions. We selected the nanoparticles known as a quantum dots due to their unique optical properties that are favored over organic dyes. This was evidenced by the fact that daphnids could be imaged for prolonged periods without loss of signal due to photo-bleaching, up to 24 hours following labeling, a task which is not possible for standard dyes used today. After one to two days on average *Daphnia* shed their carapace and thus lose their fluorescence. The fact that the quantum dots are fixed to the caparace, and that organisms like *Daphnia* molt so frequently, may introduce problems when working with natural populations. Since individuals are not synchronic, some of them might molt soon after the quantum dots were fixed, so the number of tracked individuals will be reduced within a few days. However, this would not be a problem if the number of marked individuals is sufficiently large so to compensate for this loss.

**Figure 4:**
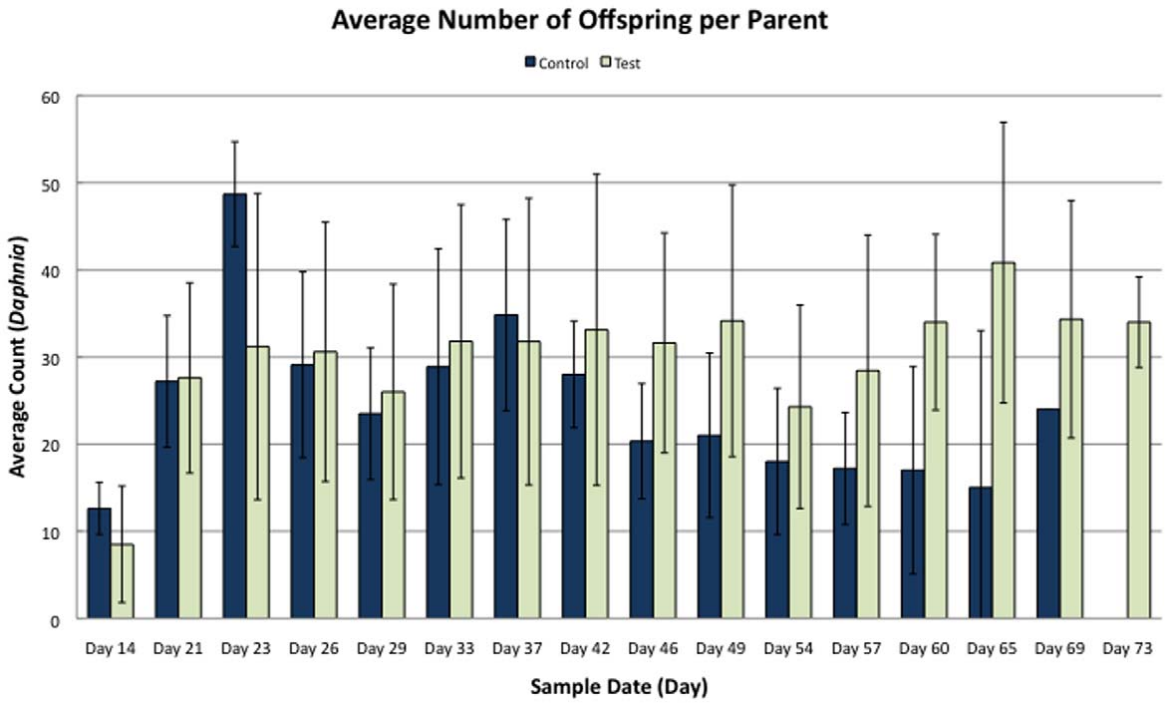
Reproductive rates in control and test groups of the biocompatibility assay, showing an average clutch size for the daphnids throughout the experiment. Day 0 is first day of life.

**Figure 5:**
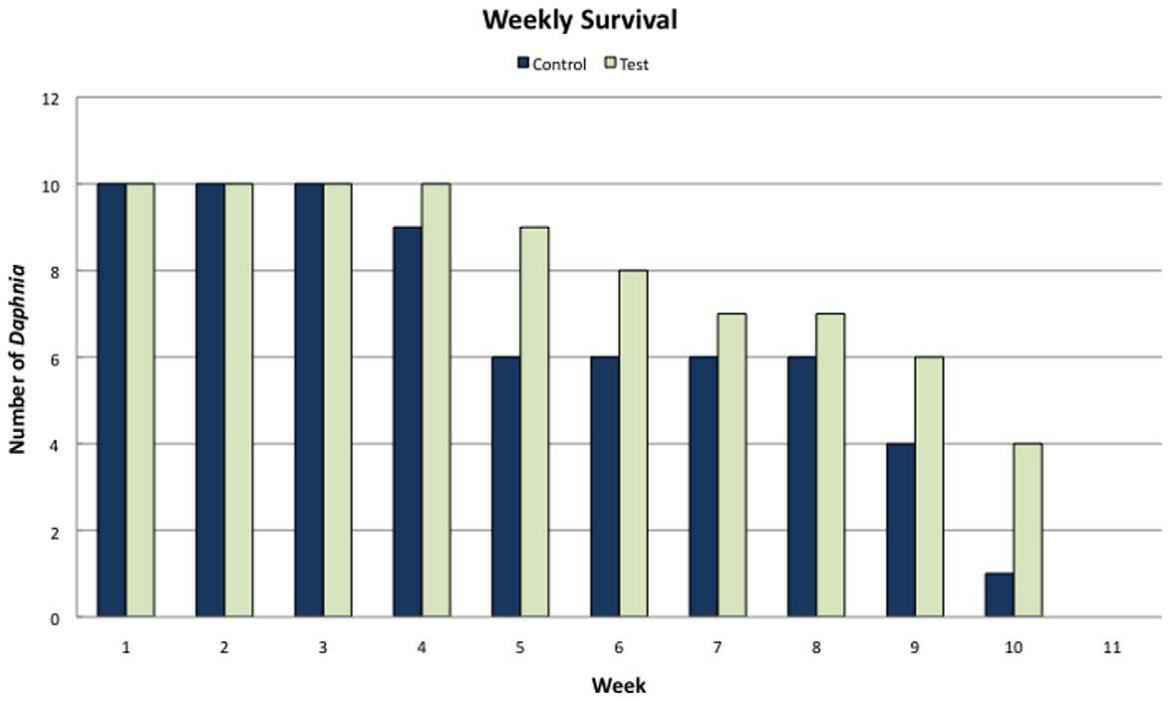
Survival in the control and test groups. Control animals were subject to the same handling and isolation treatments as the test group, but received no chemicals or quantum dots. The test group was treated with biotin and QD-SA conjugates exactly as daphnids were when imaging was performed.

Biocompatibility tests showed no significant difference in the behavior, reproduction, or survival in the *Daphnia* treated with quantum dots versus those not treated. Although no formal behavioral test was performed, the animals were checked daily for more than 10 weeks and no differences in behavior between treatments were ever recorded. The average reproductive rate did not differ between control and test groups (t = 0.70; p>0.49, Students t-test), although, surprisingly, the test group maintained a tendency for a higher average reproduction rate (Fig. 4). By the end of eleven weeks the overall survival rates from each of the two groups in the assay were comparatively similar (t = 1.10; p>0.28; Students t-test; Fig. 5), although test animals, as was seen in reproductive output, showed a tendency for a slightly longer survival than the control animals (maximum age 73 days). Hence, despite the fact that *Daphnia* are delicate organisms that are sensitive to environmental changes, they showed no signs of being negatively affected by the labeling, neither with respect to mortality nor to reproductive rates.

Looking towards our future perspectives, we have investigated the possibility to track other smaller sized aquatic animals and according to our preliminary results (data not shown) we predict that tracking is also possible for organisms smaller than one millimeter. Furthermore, more advanced cameras are available on the market which will allow for e.g. a larger depth of field (>1 m), thereby allowing for a considerable increase in study volume, and, in a future perspective, also studies in semi-natural conditions, e.g. in enclosures.

In conclusion, our study shows that nano-sized labels, not affecting behavior or life-history, can be used for routinely tracking individual animals less than 2 mm in size. We foresee that this possibility will allow tests of hypotheses and performance of studies that have never before been possible to address.
